# Resolving Scottish paediatric end-of-life conflicts

**DOI:** 10.1177/00258172221135416

**Published:** 2022-12-12

**Authors:** Sarah Sivers, Margaret Downie

**Affiliations:** The Law School, Robert Gordon University, Aberdeen

**Keywords:** Dispute resolution, paediatric end-of-life decision-making, mediation, legal framework

## Abstract

This article considers mediation as a means of resolving decision-making disputes between clinicians and parents in paediatric end-of-life cases. It examines the legal tests applied in England and Wales and notes the lack of precedent in Scotland. The advantages, disadvantages and the most appropriate style of mediation are analysed. The conclusion reached is that whilst mediation offers benefits over litigation, mediation in its current form is not necessarily the ideal dispute resolution method in such cases. For it to be so, a legal and governance framework will be required.

## Resolving Scottish paediatric end-of-life conflicts

Although not commonplace, there are disputes between parents and treating clinical teams over the continuation or withdrawal of treatment from children with life-limiting conditions. Cases like those of Charlie Gard, Archie Battersbee and more recently Baby A make the tensions and difficulties in these cases evident. They are both inherent and, to some extent, fairly self-explanatory. Parents are beyond desperate to do anything which might offer some possible hope of improvement in their child's condition while clinical teams must act in the best interests of the child when further treatment is no longer a benefit to their patient. This poses challenges when positions become entrenched.

The facts of these cases vary but the core issue remains the same; whether as a result of a rare mitochondrial condition (Gard), lack of oxygen from a ligature (Battersbee) or a catastrophic brain injury (Baby A), the patient in all three cases is in a medically futile position, but the parents of all three patients fight for either continued and/or experimental treatment, more time on life support, or to be allowed to take them home to die with family. From a legal perspective, however, the best interests test has long been accepted as the gold standard; the court takes a holistic view,^1^ going beyond the medical issues, and balancing a wide range of welfare and other factors to determine what is in the patient’s best interests.^2^ If the court determines that treatment is no longer in the patient’s best interests, then it is no longer lawful for clinicians to deliver that treatment.^3^ Recourse to the courts in the face of intractable disputes becomes inevitable. However, using the court as a forum for resolving such disputes is far from ideal. Court procedures are not swift and often come with a circus of media attention which can have significant negative impacts on both parents and clinical teams.^4^ These negative impacts come in a number of forms. In the cases of both Gard and Battersbee the level of social media interest and commentary created an environment at least as full of misinformation as it was of information, often including an unhelpful degree of morbid interest in the tragedy surrounding the family. In both cases the appeal processes resulted in a protracted succession of court hearings. The time taken also has a negative impact on the patient at the centre of the case as the clinical team can only continue the *status quo* until the final outcome of the litigation. If the court finds the treatment is no longer in the patient’s best interests, then the patient has been treated for longer than was appropriate or lawful.

Admittedly, for some individuals, a “day in court” is the only satisfactory outcome. However, other resolution processes could offer a more speedy outcome, and avoid the potential distress involved in both hearing and giving evidence on such personal and sensitive issues. A more formalised dispute resolution mechanism could potentially deliver better outcomes for some families and clinicians. Perhaps with this in mind, the judge in the English case of *Gard*^5^ was explicit in his plea for parties to use some form of mediation to either achieve resolution, or to facilitate better understanding between the parties. However, Scottish approaches to mediation have distinct differences to those in England and Wales.

## Court-based mediation in England and Wales compared to Scotland

Attitudes towards mediation in Scotland, where the Gill Report was vehemently opposed to mandatory mediation,^6^ have differed from the response in England and Wales, where the Woolf report favoured cost penalties for parties who refused to mediate.^7^ This difference in approach in each jurisdiction can still be seen in more recent reviews on the desirability and legality of widespread court-based compulsory mediation. In England and Wales, the Civil Justice Council Compulsory ADR report in 2021 concluded that parties can lawfully be compelled to mediate.^8^ This was followed by a call for evidence^9^ and a consultation by the Ministry of Justice.^10^ The proposals for consultation are for automatic compulsory mediation in small claims cases and the extension of this requirement to county court cases. The main reasons being cited are the low uptake of voluntary mediation, that mediation would be “swifter and less stressful”^10^ for the parties and that judicial time and expense should not be spent on cases where it might not be required.

In Scotland an expert group set up by Scottish Mediation reported in 2019. Its recommendation included a presumption to attend a mediation session prior to commencing litigation, the establishment of a case management function within an “Early Dispute Resolution Office”, a recommendation for a Mediation Act and the “normalising” of mediation within the civil justice system. It stopped short of recommending compulsion. The Scottish government launched a public consultation in 2020, which met with a mixed response. In the meantime, the Mediation (Scotland) Bill 2019 was introduced into the Scottish Parliament. The Bill ran out of parliamentary time, but it indicated that there seems to be the political will to embrace mediation and it is possible a form of this it will be introduced in some types of case in the near future.

## The benefits of mediation in resolving paediatric end-of-life disputes

The benefits of mediation are well-rehearsed and include speed, cost-efficiency, flexibility of outcome, and improved relationships between the parties. Litigation is a lengthy, costly method of resolving these disputes and often leads to the breakdown of relationships between clinicians and parents (as seen from the noted cases above). Mediation as the preferred solution is therefore interesting; however, it is not the only possible approach. In a literature review in 2018, Austin^11^ concluded that, while the literature supports the view that court proceedings are not the appropriate channel for such disputes, there is insufficient data on the other mechanisms to be able to determine a more appropriate solution.^11^

The litigation process has a limited range of outcomes usually resulting in a winner and a loser. The court’s interpretation of the best interests of the child, as set out above, will determine the outcome. However, very little regard is given to the impact that the breakdown of the relationship between the parties has on the interests of that child. A major benefit of mediation is that it encourages the parties to communicate with each other, thereby improving their relationship and encouraging them to generate their own, more flexible solutions.^12^ The mediator, as a neutral and unbiased third party, facilitates that process, which is informal, fast and less costly than litigation.^13^ For these reasons, mediation has generally been considered an effective means of resolving disputes and it is perhaps these advantages which the judge in *Gard* had in mind when he suggested mediation would help the parties achieve a greater understanding of each other’s positions.

## The difficulties of using mediation in this context

### The style and background of the mediator

Despite these benefits, the suitability of mediation in paediatric end-of-life cases is a complex issue. The judge in *Gard* referred to mediation broadly. However, this term covers a wide range of styles from facilitative and evaluative, to transformative. Facilitative and evaluative models focus on the dispute as a problem to be solved.^14^ In facilitative mediation, the mediator encourages communication between the parties, allowing them to generate their own solutions, while in evaluative mediation the mediator is more involved and expresses views whilst encouraging the parties to reach a solution. Transformative models focus on improving the relationship between the parties with the resolution of the dispute following on as a natural consequence,^15^ and leave responsibility for the outcome to the parties.^16^ In paediatric cases the style of mediation must be selected carefully. These cases involve complex issues which, rather than a facilitative approach, might benefit from an evaluative approach where the mediator is more directive, pointing out issues with the parties’ cases and recommending a suitable settlement.

### Reconciling mediated compromises and the best interests of the child

The role of mediation in this area is largely untried but it has been used successfully to resolve other types of medical disputes, often in the field of medical negligence claims where the primary areas of dispute are liability and compensation; it is often possible to achieve a compromise between the parties’ positions and reach a financial settlement without admitting fault. However, paediatric end-of-life cases involve complex ethical and legal principles. This is an area where Scots judicial precedent is lacking and the confidentiality of the mediation process will not assist in developing precedents. The overriding legal principle of acting in the best interests of the child cannot be abandoned, and therefore it may be difficult to reconcile a mediated compromise between the parties while simultaneously upholding the best interests of the child.

### Resolving cultural differences or power imbalances

Some of the issues relating to neutrality and bias revealed by research into mediation^17^ are particularly relevant to paediatric end-of-life disputes. Although these cases are complex and expert knowledge may be considered helpful, using a medical professional as a mediator may not be seen as neutral. Cultural and gender differences^18^ can also impact on the parties’ ability to engage with mediation and this may result in a reluctance to mediate.^19^ A key part of the mediator’s job is to “power balance”.^20^ If not performed skilfully, mediation may result in institutional bias resulting in some sections of the community faring less well than others in the mediation process. A skilled mediator would make allowance for such issues and mediation practice should be sensitively designed around the needs of the individual party.^21^ In order to ensure this, the neutrality and training of the mediator is crucial.

### Regulation of mediators

The gradual absorption of mediation into the mainstream raises questions of standards, and access to justice. In order to make the process equally available to all families, the cost of mediation in these cases should be borne by the State. In order to eliminate biases and to address complex issues, the standard of mediation is crucial and must be ensured. While solicitors are held to standards of competency and currency by their Law Society, mediators are not subject to quality control measures, as was noted in responses to the Ministry of Justice Call for Evidence. In each jurisdiction there are several professional organisations which represent mediators, provide training and maintain a register (Chartered Institute of Arbitrators, Centre for Effective Dispute Resolution and the Scottish Mediation Network) but it remains possible to practise without any qualification or training at all.

## Conclusion

Scotland and England and Wales have historically different attitudes to compulsory court-based mediation but both recognise its benefits. Mediation is an intuitively attractive solution to paediatric end-of-life disputes because of the opportunity for the parties to better understand each other’s views. However, given the fundamental rights involved, the gravity of the outcome, and the difficulties we have outlined above, mediation must be used with caution. In particular in Scotland it must be recognised that widespread mediation may hinder development of legal precedent in this area. Consideration must be given to the style of mediation to be used, and the mediation process must be carefully designed and quality assured. There must be a legal framework to determine when it is voluntary or mandatory, to guarantee separate representation for the child and to enshrine an overriding principle that the mediated settlement must be in the best interests of the child. The mediator must be trained and experienced to ensure that the parties are placed on an equal footing despite any potential disparities. There should be regulation of mediators, including standards of practice, mandatory training and a register of qualified mediators. With such safeguards in place, the adoption of mediation for any future paediatric end-of-life disputes in Scotland should be welcomed.
